# Cyanotoxins at low doses induce apoptosis and inflammatory effects in murine brain cells: Potential implications for neurodegenerative diseases

**DOI:** 10.1016/j.toxrep.2015.12.008

**Published:** 2016-01-15

**Authors:** Larissa Takser, Nora Benachour, Barry Husk, Hubert Cabana, Denis Gris

**Affiliations:** a Department of Pediatrics, Faculty of Medicine and Health Sciences, University of Sherbrooke, Sherbrooke, J1H 5N4 Quebec, Canada; b BlueLeaf Inc., 310 Chapleau Street, Drummondville, J2B 5E9 Quebec, Canada; c Environmental Engineering Laboratory, Department of Civil Engineering, University of Sherbrooke, Sherbrooke, J1K 2R1 Quebec, Canada

**Keywords:** Cyanotoxins, Low doses, Apoptosis, Inflammation, Brain cells, Neurodegenerative diseases

## Abstract

Cyanotoxins have been shown to be highly toxic for mammalian cells, including brain cells. However, little is known about their effect on inflammatory pathways. This study investigated whether mammalian brain and immune cells can be a target of certain cyanotoxins, at doses approximating those in the guideline levels for drinking water, either alone or in mixtures. We examined the effects on cellular viability, apoptosis and inflammation signalling of several toxins on murine macrophage-like RAW264.7, microglial BV-2 and neuroblastoma N2a cell lines. We tested cylindrospermopsin (CYN), microcystin-LR (MC-LR), and anatoxin-a (ATX-a), individually as well as their mixture. In addition, we studied the neurotoxins β-*N*-methylamino-l-alanine (BMAA) and its isomer 2,4-diaminobutyric acid (DAB), as well as the mixture of both. Cellular viability was determined by the MTT assay. Apoptosis induction was assessed by measuring the activation of caspases 3/7. Cell death and inflammation are the hallmarks of neurodegenerative diseases. Thus, our final step was to quantify the expression of a major proinflammatory cytokine TNF-α by ELISA. Our results show that CYN, MC-LR and ATX-a, but not BMAA and DAB, at low doses, especially when present in a mixture at threefold less concentrations than individual compounds are 3–15 times more potent at inducing apoptosis and inflammation. Our results suggest that common cyanotoxins at low doses have a potential to induce inflammation and apoptosis in immune and brain cells. Further research of the neuroinflammatory effects of these compounds in vivo is needed to improve safety limit levels for cyanotoxins in drinking water and food.

## 1 Introduction

The quality of drinking water is a major worldwide public health issue. A large number of man-made chemicals and naturally occurring toxins have been detected in many drinking water sources. Freshwater cyanobacteria may accumulate in surface water supplies as “blooms” and may concentrate on the surface as blue–green “scums”. They produce a range of toxic compounds called cyanotoxins that have a deleterious effect on raw water sources [53].

Cyanobacterial blooms are occurring with increasing frequency worldwide in fresh, brackish and marine water and in North America, Europe, Australia, Asia, Africa, Indian and Pacific oceans [10], [14], [1], [49], [9], [35]. For instance, over the last decade, in Quebec (Canada), the number of affected water bodies increased from 34 in 2004 to 119 in 2009 [36], [37] and is expected to continue to increase. Risk management of emerging cyanotoxins in worldwide water sources is compromised due to insufficient human and experimental data [11]. And, it is clear that current water quality guidelines, which focus on individual toxins, do not consider the potential combined or synergistic effects of mixtures of toxins.

Cyanotoxins are resistant to chemical and biological degradation and bioaccumulate in the food chain [32], [25]. They can be classified into three main types according to their mechanism of action such as hepatotoxins (e.g., microcystins, cylindrospermopsin), neurotoxins (e.g., anatoxins), dermatotoxins or skin irritants. Both hepatotoxins and neurotoxins are produced by cyanobacteria commonly found in surface water and therefore are of relevance to drinking water supplies [57], [10]. Thus, our interest in this study is to focus on these cyanotoxins (see Table 1). We believe that the classification of cyanotoxins by target organ (i.e., hepatotoxins or neurotoxins) is clearly not up to date, given the available knowledge on their toxic mechanisms, which are relevant for several cell types.

**Table 1:** Cyanotoxins used in this study (abbreviations, molecular formulas, and brief descriptions), which are known to act as hepatotoxins or neurotoxins.

Group of cyanotoxin	Name (abbreviation)	Chemical and molecular formula	Description
Group A	Cylindrospermopsin (CYN)	C_15_H_21_N_5_O_7_S (415.43 g/mol)	CYN is a zwitterionic tricyclic alkaloid produced by a variety of freshwater cyanobacteria. It is a protein synthesis inhibitor, may be carcinogenic, and inhibits pyrimidine nucleotide synthesis. It is classified as a cytotoxin and hepatotoxin
	Microcystin-LR (MC-LR)	C_49_H_74_N_10_O_12_ (995.2 g/mol)	MC-LR is a cyclic heptapeptide produced by cyanobacteria. It is considered to be the most toxic compound of the microcystin family. It is a tumor promoter and selective inhibitor of protein phosphatase 1 (PP1) and 2A (PP2A). It is classified as a hepatotoxin
	Anatoxin-a (ATX-a)	C_10_H_15_NO (281.3 g/mol)	ATX-a, also known as Very Fast Death Factor, is a secondary, bicyclic amine alkaloid and cyanotoxin with acute neurotoxicity. It was first discovered in the early 1960s in Canada, and was isolated in 1972. It is a nicotinic acetylcholine receptor ligand

Group B	β-*N*-methylamino-l-alanine (BMAA)	C_4_H_10_N_2_O_2_ (154.60 g/mol)	BMAA is a non-proteinogenic amino acid produced by cyanobacteria and cycads. It is considered a possible cause of ALS/PDC (amyotrophic lateral sclerosis/parkinsonism–dementia complex) and its toxicity has been associated with possible chronic neurodegeneration. It is classified as a neurotoxin
	2,4-Diaminobutyric acid (DAB)	C_4_H_10_N_2_O_2_ (191.06 g/mol)	DAB is a structural isomer of BMAA and is classified as a neurotoxin.

The hepatotoxin, microcystin-LR (MC-LR), was the first microcystin chemically identified and the most studied to date. It has been associated with most of the incidents of toxicity involving microcystins in several countries [10], [25]. MC-LR is inhibitor of protein phosphatases 1 and 2A, which are present in virtually all cells and involved in several cell processes including cell division and apoptosis, induces sustained phosphorylation of proteins in cells and DNA damage, and is a potent tumor promoter in chronic exposures [21], [49].

The tricyclic guanidinic alkaloid cylindrospermopsin (CYN) is also classified as a hepatotoxin but has a different mechanism of action, being a protein and glutathione synthesis inhibitor, with a major impact on liver cells. Indeed, CYN or its metabolite(s) have been shown in vivo to form adducts in liver DNA in mice [47], [20], [22]. The binding of cylindrospermopsin or a metabolite to DNA, and possibly to RNA, in liver cells has been proposed as a possible mechanism for the inhibition of protein synthesis that occurs with cylindrospermopsin toxicity but also in other organs such as kidneys, spleen, intestine, thymus and heart in vertebrates [13], [14]. In contrast to neurotoxic alkaloids, CYN acts more slowly, taking about 5–6 days to kill mice with a LD50 of 200 μg/kg [12]. It has caused serious gastrointestinal and respiratory distress through exposure via drinking water in humans and domestic animals [8], [14].

In general, the members of the neurotoxin category are not considered to widespread as hepatotoxins in water supplies, and they are not considered to pose the same degree of risk from chronic exposure as microcystins [57]. Anatoxin-a (ATX-a) was the first cyanotoxin belonging to the class of the neurotoxins to be characterized [7]. This alkaloid is a potent post-synaptic cholinergic nicotinic agonist and neuromuscular blocking agent that is a highly toxic nerve poison but has a short biological half-life [12]. The Guideline value (GV) for drinking water was established at 12.24 μg/l [18].

β-*N*-methylamino-l-alanine (BMAA) has been detected in several resource waterbodies [38] and was the first neurotoxin isolated from cycad trees on Guam in 1967. It may be involved in multiple neurodegenerative diseases [16] such as amyotrophic lateral sclerosis/parkinsonism–dementia complex (ALS/PDC) on the Island of Guam in the East Pacific [3]. BMAA has also been detected in the brains of Canadian patients with Alzheimer’s disease [41]; however, others have failed to detect BMAA in brains of patients with neurodegenerative diseases [39]. BMAA has an structural isomer, the 2,4-diaminobutyric acid (DAB) which is very similar to the non-essential amino acid alanine.

It is well known that neurodegenerative diseases, such as Parkinson’s and Alzheimer’s diseases are associated with inflammation processes [31]. Inflammation is a rapid response of tissue to injury and is characterized in the acute phase by increased blood flow and vascular permeability along with the accumulation of fluid, leucocytes (monocytes, macrophages), and inflammatory mediators, such as cytokines TNF-α, IL-6, IL-1 [24], [48]. The high levels of Tumor necrosis factor alpha (TNF-α), under these conditions, result in hypersensitivity reactions following by chronic inflammation [33], [48]. Recent studies show that the brain can be a key target for certain cyanotoxins such as MCs and BMAA [25], [26], [42]. Their potential implication in neurodegenerative diseases such as Alzheimer’s disease has been suggested [42].

Given that several cyanotoxins may be present in drinking water at the same time, there is no data on their effects in mixtures. The aim of this study was to test the hypothesis that cyanotoxins at low doses are neurotoxic when applied in environmentally relevant mixtures (e.g., BMAA and DAB co-occur in water [19]; microcystin, ATX-a, and CYN producing cyanobacteria co-occur in sources of drinking water [51]). Thus, we examined cellular viability, apoptosis and the pro-inflammatory effects of each cyanotoxin listed in Table 1, alone or in mixtures. We hypothesized that these cyanotoxins at doses, which we estimated close to those presumed to be safe (between a no-observed-adverse-effect level (NOAEL) and guideline values (GV) in drinking water) will induce a pro-inflammatory response in brain and immune cells, when administered in mixture. Until now, NOAEL and GV are available only for MC-LR, CYN and ATX-a (see Table 2). In addition, GV for cyanotoxins in drinking water vary among countries and there are insufficient data to derive a GV for all cyanobacterial toxins [17], [5]. The selection of doses to test was based on both, the presumption that these doses will not induce acute cell toxicity (based on available publications and below known LD50 values) and/or the concentration close to the GV (Table 3). Given that GV are derived by application of an uncertainty factor (usually 1000), we estimated that concentrations which vary within a 1000 fold interval above the GV do not exceed NOAEL levels.

**Table 2:** Tolerable daily intake (TDI), acute toxicity (LD50), and guideline values (GV) in drinking water, for cyanotoxins used in this study.

Cyanotoxin	Oral toxicity test μg/kg(b.w.)/day	TDI^f^ μg/kg(b.w.)/day	LD50^a^ μg/kg(b.w.) Intra-peritoneal mouse	GV^b^ μg/l drinking water	References
CYN	NOAEL = 30^c^ (mouse, 90 days)	0.03	200–2100	1 or 15	[22], [5]
MC-LR	NOAEL^c^ = 40 (mouse, 13 weeks)	0.04	25 to ∼1000	1 or 1.5	[23], [57], [27]
	LOAEL^d^ = 100 (pig, 44 days)	0.067			
ATX-a	NOAEL^c^ = 510 (mouse, 7 weeks)	0.51	250	12.24	[2], [18]
BMAA	ND	ND	EC_50_^e^ about 1 mM (118 μg/ml, in cells)	ND	[55], [45]
DAB	ND	ND	ND	ND	/

a LD50: median lethal dose of a substance, or the amount required to kill 50% of a given test population.

b GV: estimates made by assuming a human body wt of 60 kg; allocation factor, AF, of 0.8 and daily drinking water consumption, *C*, of 2 l. A guideline value (GV; μg/l water), to be used in formulating risk management strategies to strengthen drinking water safety throughout lifetime consumption, can be calculated as: GV = (TDI × body wt × AF)/*C*; where body wt is assumed to be 60 kg for a human adult and AF is the allocation factor: the proportion of the TDI via drinking water. Because some oral exposure may be via food or dietary supplements [13], an AF of 0.8 (80% of total intake) is assumed for drinking water and *C* = drinking water consumption per day, assumed to be 2 l for an adult. It should be noted that the values for GV in Table 2 were taken from the literature and were not calculated from the TDI values. In actual fact, the values for GV in the table are very different from those resulting from the use of the formula given above.

c NOAEL: no-observed-adverse-effect-level.

d LOAEL: lowest-observed-adverse-effect-level.

e EC_50_: effective concentration for 50% of organisms tested. ND: no data.

f TDI: tolerable daily intake is derived by applying an uncertainty factor of 1000 to the NOAEL. ND: no data.

**Table 3:** Comparison of cyanotoxin concentrations used in our experiment with the GV values presented in Table 2.

Treatment	Dose (μM)	Group A (μg/L)			Group B (μg/L)		Comparison with GV for CYN, MC-LR, and ATX-a (see Table 2)
		CYN	MC-LR	ATX-a	BMAA	DAB	
Alone	0.001	0.415	0.995	0.281	0.154	0.191	≤GV
	0.1	41.43	99.52	28.13	15.46	19.11	>GV
	10	4154	9952	2813	1546	1911	>GV

Equimolar mixtures Mix A (÷3) Mix B (÷2)	0.001	0.138	0.332	0.094	0.077	0.095	<GV
	0.1	13.8	33.2	9.4	7.7	9.5	≥GV
	10	1384.8	3317.3	937.7	773.0	955.5	>GV

We used murine macrophage-like RAW264.7, microglial BV-2, and neuroblastoma N2a cell lines as these cell lines are frequently used models to study neurotoxicity and brain damage resulting from inflammation [28], [54], [15].

## 2 Materials and methods

### 2.1 Chemicals and reagents

Cyanotoxins used in this work were used alone and in two mixtures (group A and group B). See Table 1 for chemical formulas and for the brief description of each toxin. CYN, MC-LR, and ATX-a were purchased from Enzo Life Sciences, Inc. (NY, USA). MC-LR and CYN were dissolved in sterile water to 1 mM. ATX-a was dissolved in sterile water to 2.2 mM. BMAA and DAB were obtained from Sigma–Aldrich Canada Co. (ON, Canada) and dissolved in sterile water to 65 mM. All solutions were stored at −80 °C until use. 3-(4,5-Dimethylthiazol-2-yl)-2,5-diphenyl tetrazolium bromide (MTT) was obtained from Sigma–Aldrich Canada Cie. (ON, Canada). MTT was prepared as a 5 mg/ml stock solution in phosphate-buffered saline (PBS 1X), filtered through a 0.22 μm filter before use, and diluted to 1 mg/ml in a serum-containing medium. Mouse TNF-α ELISA assay kit was purchased from Thermo Fisher Scientific Company (ON, Canada). Caspase-Glo 3/7Aassay kit was purchased from Promega Corporation (WI, USA). All other compounds and reagents, were obtained from Wisent, Inc. (QC, Canada).

### 2.2 Cell cultures

RAW246.7 murine macrophage-like cell line has been previously used to study inflammation-induced neurodegeneration, toxicology, and immunity [31], [15]. These cells were maintained in Dulbecco’s modified Eagle’s medium (DMEM).

BV-2 cell line represents immortalised murine microglial cells that are often used to study mechanisms of microglial-dependent neuroinflammation [28]. These cells were maintained in the Roswell Park Memorial Institute (RPMI-1640) medium.

N2a or Neuro 2A is a mouse neurblastoma-derived cell line that has been used to study neuronal differentiation, axonal growth and signalling pathways [52]. N2a cells were given as a gift by Dr. Pavel Gris and were maintained in Dulbecco’s modified Eagle’s medium/Ham’s F12 (DMEM/F12).

Culture mediums were supplemented with 10% foetal bovine serum (FBS), 1% nonessential amino acids (100X), 1% sodium pyruvate (100 mM), 1% l-glutamine, and 1% penicillin/streptomycin (100X) before use. All cell lines were kept at 37 °C in a 5% CO_2_ humidified incubator for 80% of confluence.

### 2.3 Cell treatments

The three cell lines were exposed to low doses (0.001, 0.1 and 10 μM) of each cyanotoxin, cited in Table 1, alone or in mixture for various times: 24, 48, and 72 h. We have combined them into two groups: Group A contains CYN, MC-LR, ATX-a and their equimolar 10 μM mixture (Mix A) (e.g., 3.3/3.3/3.3 μM); Group B includes BMAA, DAB and their 10 μM equimolar mixture (Mix B). Doses of each cyanotoxin in this study were around guideline values (GV) in drinking water, tolerable daily intake (TDI) or no-observed-adverse-effect-level (NOAEL) as described in Table 2, Table 3.

### 2.4 Cell death and apoptotic assessments

#### 2.4.1 Cellular viability

Cellular viability was performed and quantified by MTT assay as previously described [4]. This method measures mitochondrial enzyme succinate dehydrogenase (SD) activity. Inhibition of SD is an indicator of cell death. The assay is based on the cleavage of MTT into a blue coloured product (formazan) by SD [40]. After cell treatments, at different times 24, 48, and 72 h, adherent cells were washed and incubated MTT for 1 h at 37 °C and 0.04 *N*-hydrochloric acid-containing isopropanol solution was added to each well. The optical density (OD) was measured at 570 nm wavelength and the reference measurement at 720 nm wavelength using a multimode microplate reader Nanoquant Infinite M200 (Tecan US, Inc., NC, USA). Results are presented as % in comparison to the non treated cells (control = 100%).

#### 2.4.2 Apoptotic cell death

Apoptotic cell death was measured with Caspase-Glo^®^ 3/7 assay (Promega Corporation, WI, USA). It is a luminescent kit designed for automated high-throughput screening of caspase-3 and -7 activities in purified enzyme preparations or cultures of adherent or suspension cells [44]. The assay provides a pro-luminescent caspase-3/7 substrate, which contains the tetrapeptide sequence DEVD. When this substrate is cleaved, amino-luciferin is released, a substrate of luciferase used in the production of light. The Caspase-Glo^®^ 3/7 bioassay was carried out in 96-well white plates. Cell cultures were exposed for 24 h to different concentrations of cyanotoxins of Group A or B. Luminescence was measured at 565 nm using the Nanoquant Infinite M200 microplate luminometer reader. Flow cytometry was used to analyses percentage of apoptotic and necrotic cells by annexin V PI staining as previously described [29]. ROS level was evaluated as previously described [30]. Samples were analyzed by flow cytometry using a FACS Calibur. Data were analyzed using FlowJo software.

### 2.5 Inflammation assessment

#### 2.5.1 TNF-α measurement

TNF-α was measured using an Enzyme Linked ImmunoSorbent Assay (ELISA) (R&D Systems Inc., MN, USA) according to manufacturer specification. Quantitative TNF-α protein concentration was determined in cell culture supernatant of each cell type after 24 h of exposure with the different doses of cyanotoxins played alone or in the combination of Group A or Group B. The monoclonal antibody specific for mice TNF-α had been pre-coated onto a microplate. Standards, controls and samples are pipetted into the wells and any TNF-α present is bound by the immobilized antibody. After washing away any unbound substances, an enzyme-linked polyclonal antibody specific for TNF-α is added and incubated for 2 h at room temperature on the shaker. Following a wash to remove any unbound antibody-enzyme reagent, an enhanced luminal/peroxide substrate solution is added to the wells and incubated for 20 min then light is produced in proportion to the amount of TNF-α bound in the initial step. The optical density (OD) was measured at wavelength of 450 nm and the reference measurement at wavelength of 550 nm using a multimode microplate reader Nanoquant Infinite M200.

### 2.6 Statistical analysis

The experiments were repeated four times on independent cell cultures. All data were presented as the mean ± standard error of mean (SEM). Statistical analyses were performed using GraphPad Software Prism 6.0 (GraphPad Software, CA, USA). Differences between groups in comparison with control were analyzed using a nonparametric Mann–Whitney test and one-way ANOVA test. The differences were considered significant at *p* values less than 0.05.

## 3 Results

### 3.1 MTT assay

**Fig. 1:**
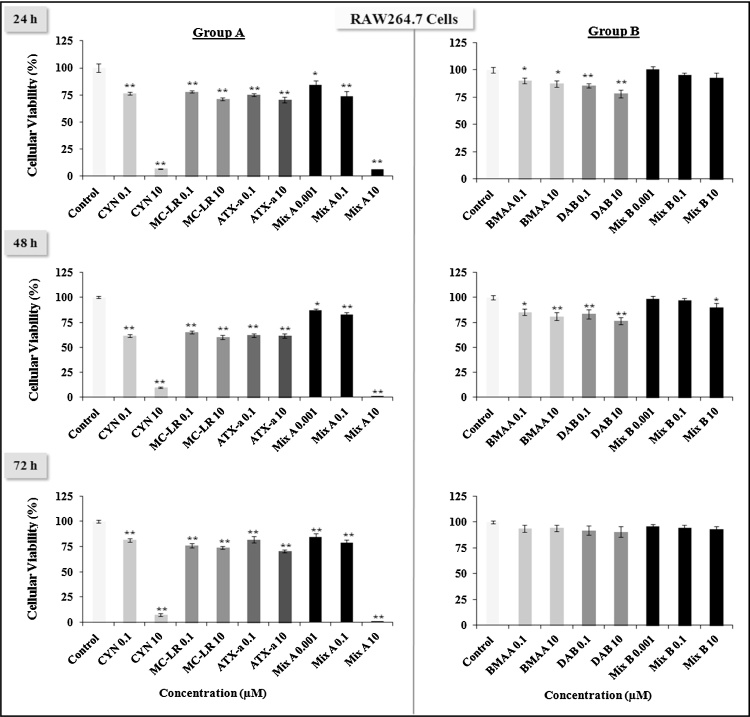
Effects of low doses (0.001, 0.1, 10 μM) of cyanotoxins alone or in combination from group A (CYN, MC-LR, ATX-a, Mix A) and group B (BMAA, DAB, Mix B) on the murine macrophage-like RAW246.7 cell viability at various times (24, 48, 72 h) evaluated by the MTT assay. The results are presented at % of cell viability measurement (mean ± SEM) compared to non-treated cells (Control = 100%), *n* = 12, *p < 0.05 or **p < 0.001.

**Fig. 2:**
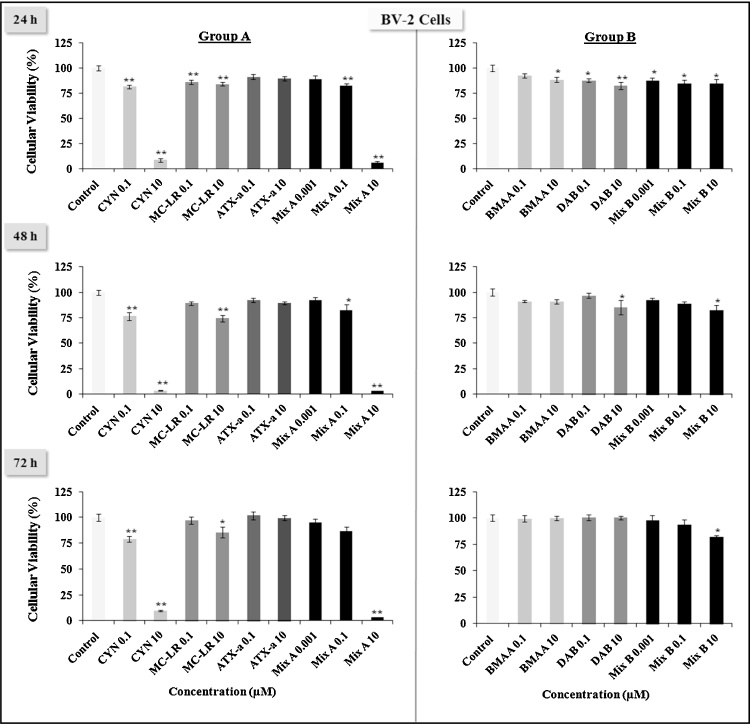
Effects of low doses (0.001, 0.1, 10 μM) of cyanotoxins alone or in combination from group A (CYN, MC-LR, ATX-a, Mix A) and group B (BMAA, DAB, Mix B) on the resident macrophage-like cells of the central nervous system (CNS) BV-2 cell viability at various times (24, 48, 72 h) evaluated by the MTT assay. The results are presented at % of cell viability measurement (mean ± SEM) compared to non-treated cells (Control = 100%), *n* = 12, *p < 0.05 or **p < 0.001.

**Fig. 3:**
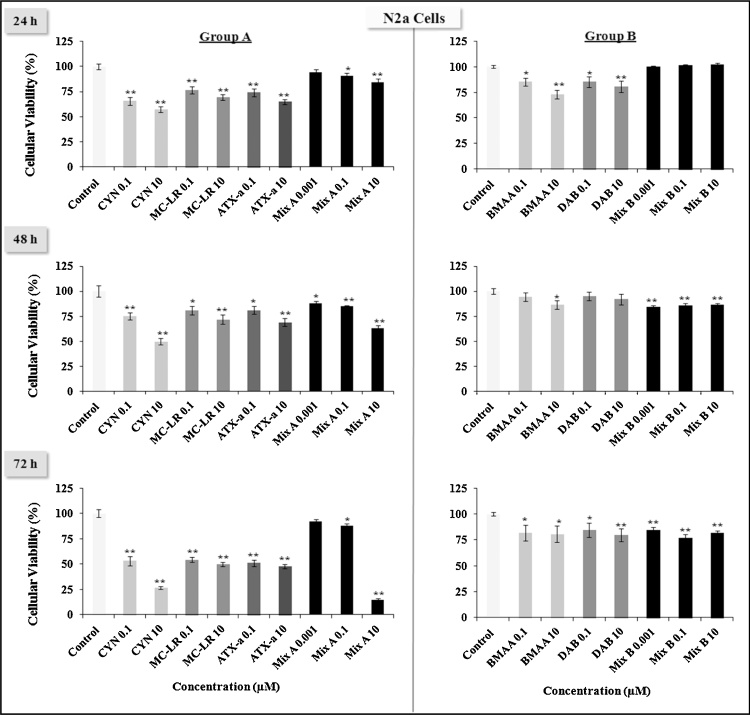
Effects of low doses (0.001, 0.1, 10 μM) of cyanotoxins alone or in combination from group A (CYN, MC-LR, ATX-a, Mix A) and group B (BMAA, DAB, Mix B) on the murine neuroblastoma N2a cell viability at various times (24, 48, 72 h) evaluated by the MTT assay. The results are presented at % of cell viability measurement (mean ± SEM) compared to non-treated cells (Control = 100%), *n* = 12, *p < 0.05 or **p < 0.001.

As shown in Fig. 1, Fig. 2, Fig. 3 , Group A toxins are more toxic than Group B in all three cell types tested. Group B toxins never reached LD50 levels of toxicity at any of the treatment times (24, 48 or 72 h). Among the three Group A toxins, MC-LR and ATX-a never reached LD50 levels of toxicity_,_ while CYN and equimolar Mix A can reach LD50 between 0.1 μM to 10 μM at the different times (24, 48 or 72 h). These doses were 5–500-fold lower than the Tolerable Daily Intake (TDI) for CYN which is 0.02 g/kg (b.w.)/day. Indeed, 10 μM of CYN or 10 μM of Mix A, which is composed of 3.3 μM of each of the Group A toxins induce almost a total death of RAW264.7 and BV-2 cells (Fig. 1, Fig. 2).

Treatment with CYN or Mix A induced cell death of the neuroblastoma N2a cell line in a dose and time dependent manner (Fig. 3). For instance, CYN reached LD50 at 10 μM at both 24 h and 48 h, but at 72 h, even the 0.1 μM showed LD50 levels of toxicity. Mix A (10 μM) reached LD50 only at 72 h. N2a cells were more sensitive to BMAA, DAB and their mixture (Mix B), and to MC-LR and ATX-a, than RAW264.7 and BV-2 cells. No LD50 level of toxicity were observed for MC-LR and ATX-a in RAW264.7 and BV-2, while in N2a at 72 h, we observed a significant decrease in cell viability with MC-LR and ATX-a, which was similar to CYN at 0.1 μM.

### 3.2 Apoptotic cell death

**Fig. 4:**
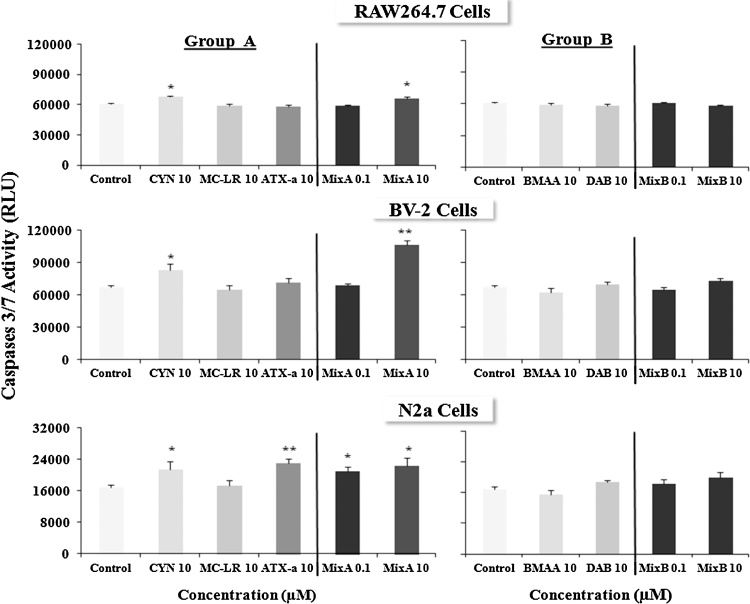
Apoptotic effects of the low doses (0.001, 0.1, 10 μM) of cyanotoxins alone or in combination from group A (CYN, MC-LR, ATX-a, Mix A) and group B (BMAA, DAB, Mix B) at 24 h through caspases 3/7 induction in three murine cell lines (RAW264.7, BV-2 and N2a). Apoptosis was tested by the Caspase-Glo^®^ 3/7 assay. The results are presented in relative units (RLU) of measurements (mean ± SEM) compared to non-treated cells (control), *n* = 16, *p < 0.05 or **p < 0.001.

Fig. 4 shows caspase 3/7 activity in RAW264.7, BV-2 and N2a cells after exposure to different toxins. Our data demonstrate that in all cell lines, BMAA, its isomer DAB and their equimolar Mix B did not induce apoptosis. In contrast, CYN and Mix A showed a clear induction of caspases, an early sign of apoptosis. RAW264.7 and BV-2 cells, 10 μM of CYN or Mix A, at 24 h, induce significant activation of caspases 3/7 (*n* = 16, *p < 0.05). In BV-2, the effect of Mix A, which contains 3.3 μM CYN, was 3–6 fold higher than compared to CYN (10 μM) alone. Furthermore, N2a cells were more sensitive to ATX-a and Mix A as compared to RAW264.7 and BV-2 cells. Indeed, both CYN at 10 μM (*n* = 16, *p < 0.05) and 10 μM of the neurotoxin ATX-a have apoptotic effects on N2a cells (*n* = 16, **p < 0.001). In N2a cells, Mix A induced apoptosis at both 0.1 μM and 10 μM (*n* = 16, *p < 0.05 for both).

**Fig. 5:**
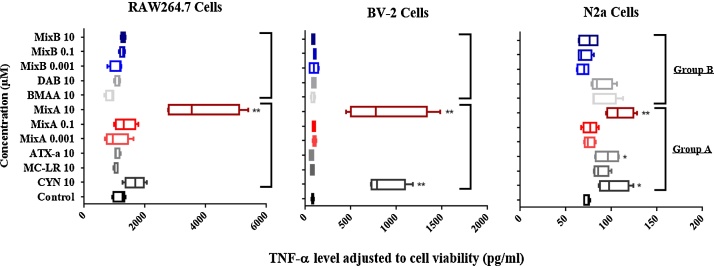
Inflammatory effects of the low doses (0.001, 0.1, 10 μM) of cyanotoxins alone or in combination from group A (CYN, MC-LR, ATX-a, Mix A) and group B (BMAA, DAB, Mix B) at 24 h on three murine cell lines (RAW264.7, BV-2 and N2a). TNF-α protein quantification was measured using ELISA method. TNF-α protein concentration (pg/ml) was adjusted to cell viability and shown as mean ± SEM compared to non-treated cells (control), *n* = 8, *p < 0.05 or **p < 0.001.

Fig. 5 shows TNF-α levels adjusted for cell viability following treatment with the toxins for 24 h. BMAA, DAB and Mix B did not affect TNF-α levels. ATX-a significantly increased TNF-α secretion only in N2a cells (*n* = 8, *p < 0.05). CYN significantly increased the TNF-α levels, in N2a cells (*n* = 8, *p < 0.05) and in BV-2 cells (*n* = 8, **p < 0.001) while Mix A increased TNF-α all these cell lines.

**Fig. 6:**
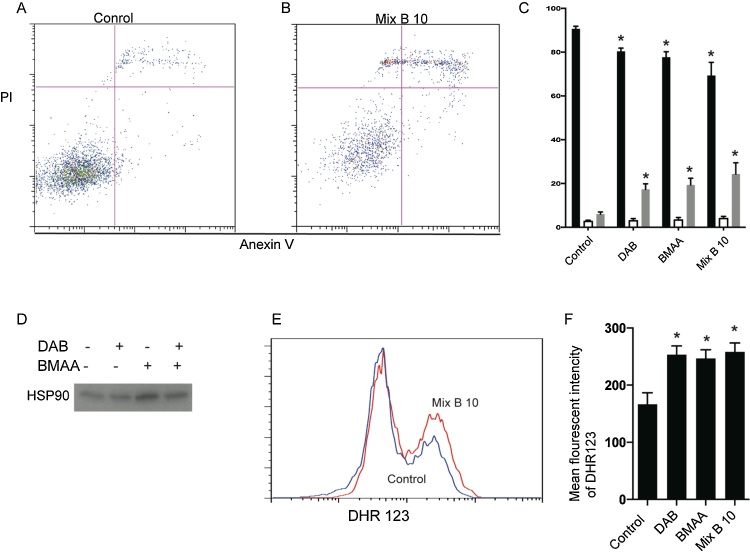
Cyanotoxins induce necrosis in neuronal cell line. N2a cell line was treated with 10 μM DAB, 10 μM BMAA and Mix B 10 (DAB + BMAA at 10 μM concentration). (A and B) are representative histograms of flow cytometry with annexin V propidium iodide (PI) of control and Mix B 10 treated cells respectively. (C) Quantification of flow cytometry defining live (black bars), apoptotic (open bars), and necrotic cells (gray bars). (D) Representative western blot showing relative amount of HSP90 in the supernatant. (E) is a representative histogram overlay showing level of fluorescence after staining with DHR123 that start to fluoresce up on oxidation by ROS. (F) Quantification of flow cytometry experiment measuring ROS production. Each experiment was repeated at least 3 times *p < 0.5, *n* = 3.

We observed significant increase in cell death in N2a cells that was not accompanied by increase in caspases activity. Looking further into the mode of cell death of N2a cells after cyanotoxin treatment, we performed flow cytometry experiments using annexin V PI staining (Fig. 6A–C). Annexin V binds to posphoserine that flips from intracellular to extracellular space at initiation of apoptosis. Thus cells in early and late stages of apoptosis will be labeled with annexin V. PI is a nucleic acid dye that penetrates necrotic cells and cells at the late stages of apoptosis. If cells are not labelled by either dye they are considered life cells. Results demonstrate that after treatment with DAB, BMAA or the combination of these two toxins there is a significant decrease in life cells and increase in cells that are double positive for annexin V and PI, while single positive annexin V cells remain constant. Looking further into the mode of cell death after cyanotoxin treatment, we performed western blotting of HSP90 in cell culture supernatants. The amount of HSP90 in supernatants is indicative of necrosis. Consistent with PI staining, we observed significantly increased level of HSP90 in supernatants of N2a cells after all treatments with cyanotoxin (Fig. 6D). Such increases in necrosis can be induced by dramatic increase in ROS level within the cells. To verify this, we stained cells with DHR123 dye that starts to fluoresce up on oxidation with ROS. We observed significant induction of ROS after all treatments (Fig. 6E,F). Interestingly, there was no difference between DAB, BMAA and mix B 10 treatments in any outcomes measured (Fig. 6A–F).

## 4 Discussion

This is the first study to test common cyanotoxins for potential neuroinflammatory effects. Our results show that cyanotoxins CYN, MC-LR and ATX were more toxic than BMAA and its isomer DAB. In addition, our results suggest that a mixture of CYN, MC-LR and ATX-a is at least 3–15 fold more potent for the induction of apoptosis and inflammation compared to the individual compounds. Our results show that the cyanotoxins tested in this study at doses that are considered to be “safe”, induce cell death and inflammation, especially in mixtures, in cultured neurons and glial cells. The presence of these toxins in the brain has already been reported and reviewed for MCs and BMAA [55], [25], [42]. However, the extrapolation of in vitro findings to in vivo and to human health effects requires knowledge of the bioavailability, the elimination of these compounds and must certainly take into account their potential bioaccumultation, metabolism and time-delayed effects, especially in the developing brain which lacks blood-brain barrier. To date, in regards to the majority of cyanotoxins, this knowledge is very limited [56], [25], [6].

The murine macrophage-like RAW246.7 cell line has been proposed as in vitro model of neurodegeneration resulting from inflammation [31], [15]. BV-2 microglia cells, which are the resident macrophage-like cells in the central nervous system, have been used to study neuroinflammation [43], [28]. Both these cell lines showed a similar pattern of response to cyanotoxins in our study. They were more affected by CYN and by the mixture of MC-LR, CYN, and ATX-a, which induced rapid cell death and TNF-α production in survived cells, than by MC-LR and ATX-a alone.

N2a cells were less resistant to MC-LR, CYN, and ATX-a in MMT assay, compared to macrophage-like cells. ATX-a alone and the mixture of cyanotoxins of Group A at dose of 0.1 μM, induced apoptotic responses which was not observed in macrophage-like cells. This finding confirms that ATX-a is a potent neurotoxin in vitro even at doses much less than established NOAEL, and that neurons are more sensitive to the mixture of these cyanotoxins. Although neurons have a limited capacity for cytokine production, our data on TNF-α in N2a cells show a significant pro-inflammatory response to CYN, ATX-a, and to the mixture at 10 μM. Our results show that low doses of CYN and ATX-a, or their combination are highly relevant for neurodegeneration.

Recently, it was reported that high concentrations of MC-LR and BMAA induce significant neuronal apoptosis acting through induction of caspase activity, chromatin condensation [26], and neurodegenerative effects, reminiscent of Alzheimer’s disease type human tauopathies [34], [42]. For instance, it has been shown that MC-LR treated hippocampi showed alterations in proteins involved in the cytoskeleton, neurodegeneration, oxidative stress, apoptosis, and energy metabolism. Three proteins related to neurodegenerative disease, septin5, α-internexin, and α-synuclein, were increased by MC-LR exposure [26]. Our data suggest that MC-LR has less potential to induce neurodegeneration.

BMAA, an analog of the amino acid alanine, has been found to be incorporated into proteins isolated from the brains of patients affected by amyotrophic lateral sclerosis [46]. In our study, BMAA, DAB and their combination (Mix B) up to 10 μM concentration, do not affect TNF-α levels or apoptosis mediated by caspases 3/7, but increased cell death by 20% in all cell lines. N2a cells cell death was not associated with increased caspase activity suggesting alternative mode of cell death. Indeed, using flow cytometry, we observed increase amount of necrotic cells after toxin treatment, while apoptotic cells did not change We measured necrosis by amount of HSP90, that is normally in the cell, in supernatants. We observed increase in necrosis in N2A cells after treatment with ether DAB or BAAM, but the effect was not additive, suggesting that both toxins affect the same pathway. Most likely such increases in necrosis are induced by significant induction in ROS production that we observed after toxin treatments. Both, BMAA and DAB increase oxidative stress, which leads to necrotic damage in neuronal cells. For instance, it is reported that BMAA induced the both pathways in rat cerebellar granule cells [50].

In conclusion, our study suggests that environmentally relevant low doses of cyanotoxins, commonly found in mixture in drinking water, induce pro-apoptotic and pro-inflammatory effects in macrophage-like and neural cells. These results have potential implication for further research on cancer and on neurodegenerative diseases, including Alzheimer’s disease in relation to increasing contamination of water bodies and food by cyanotoxins.

## Conflict of interest

All authors declare that they have no competing interests.

## Transparency document
